# Driver Face Verification with Depth Maps

**DOI:** 10.3390/s19153361

**Published:** 2019-07-31

**Authors:** Guido Borghi, Stefano Pini, Roberto Vezzani, Rita Cucchiara

**Affiliations:** Softech-ICT, Dipartimento di Ingegneria Enzo Ferrari, Università degli studi di Modena e Reggio Emilia, 41125 Modena, Italy

**Keywords:** driver face verification, depth maps, fully-convolutional network, Siamese model, deep learning, automotive

## Abstract

Face verification is the task of checking if two provided images contain the face of the same person or not. In this work, we propose a fully-convolutional Siamese architecture to tackle this task, achieving state-of-the-art results on three publicly-released datasets, namely *Pandora*, *High-Resolution Range-based Face Database* (HRRFaceD), and *CurtinFaces*. The proposed method takes depth maps as the input, since depth cameras have been proven to be more reliable in different illumination conditions. Thus, the system is able to work even in the case of the total or partial absence of external light sources, which is a key feature for automotive applications. From the algorithmic point of view, we propose a fully-convolutional architecture with a limited number of parameters, capable of dealing with the small amount of depth data available for training and able to run in real time even on a CPU and embedded boards. The experimental results show acceptable accuracy to allow exploitation in real-world applications with in-board cameras. Finally, exploiting the presence of faces occluded by various head garments and extreme head poses available in the *Pandora* dataset, we successfully test the proposed system also during strong visual occlusions. The excellent results obtained confirm the efficacy of the proposed method.

## 1. Introduction

Face recognition algorithms are traditionally split into two specific tasks by the computer vision community [1]: *face verification* and *face identification*. The former is based on a *one-to-one* comparison; given a pair of images as input, a face verification system should predict if the input items contain faces of the same person or not. Instead, the latter performs a *one-to-many* comparison; a given input face (probe) is compared with a collection of candidates (gallery) to find the best match.

Face recognition is usually applied on intensity (RGB or gray-level) [2] images that contain appearance features useful for solving the task. However, intensity data may be influenced by the illumination conditions, which can be very poor or even absent in some situations.

In this work, we investigate the face verification task applied to the automotive context, a challenging environment that imposes some particular constraints. The possibility to recognize the identity of the driver can be useful for a variety of applications. For instance, the driver–car interaction can be enhanced by adapting the infotainment content and interface to the specific driver. Moreover, driver monitoring systems can be improved in efficacy, since in-cabin monitoring algorithms can be focused on the driver, with his/her known health issues or behavior trends [3]. Finally, a reliable face verification operation could be exploited in order to improve the safety of the driving activity [4]: some functions (like speed limits or the engine power) can be associated with the age of the driver or with his/her ability. As previously mentioned, specific requirements are imposed by the automotive context.

*Light invariance* is the first key requirement, since in-board monitoring systems undergo dramatic light changes, which often occur during the everyday driving activity (for instance, due to bad weather conditions or tunnels) [3]. A second key element is the compliance with the *real-time* performance of the system, since monitoring and interaction systems have to quickly detect anomalies and promptly provide feedback and alarms. The *non-invasivity* of the in-cabin device is another fundamental feature, since all ADAS systems should not impede driver’s movements, limit the gaze, or distract the driver from the driving activity [5]. In this context, visual systems based on near-infrared acquisition devices are a worthy solution [6].

Near-infrared sensors could be affected by an intensive and direct illumination (e.g., the Sun facing the device), but they can be a powerful and safe solution in the case of a lack of sufficient external illumination, which is a common case in the driving activity during sunset, the night, or inside tunnels. Moreover, the potential interference of sun light can be reduced by placing these sensors in specific in-cabin locations.

Several solutions [7], based on *physiological signals* (electroencephalogram, electrocardiogram, and electromyogram) or *vehicle signals* (such as signals acquired through the car bus), are acquired through sensors or other hardware devices that are directly placed on the driver’s body or that require specific hardware integration with the car systems. Therefore, these methods require either the use of invasive systems or a hard integration with car hardware.

For these reasons, we propose a framework based only on depth maps computed from *Time-of-Flight* (ToF) sensors (i.e., near-infrared sensors), even though the large majority of face recognition methods available in the literature is based on intensity data. Our challenge is to obtain similar performance of state-of-the-art RGB systems, but using depth maps only, which can be reliably used even when the illumination conditions are not good enough.

The existence of very large-scale datasets containing RGB images, like *Labeled Faces in the Wild* [8], the *YouTube Faces Database* [9], *CelebA* [10], and *MS-Celeb-1M* [11], allows the training of extremely deep convolutional neural networks, such as *DeepFace* [12], *Facenet* [13], and the work of Parkhi et al. [14]. Only a few works in the literature use non-intensity images as input, like depth maps and thermal images [15,16]. These kinds of data are almost insensitive to light changes; therefore, they are suitable for challenging unconstrained environments, like the automotive field. However, only a few datasets that contain non-intensity images are currently publicly available [17,18,19,20].

A preliminary version of this work was presented in [21], where both RGB and depth cameras were used during the system training. In this paper, we improve the proposed method by completely removing the need for RGB cameras and proposing a new network architecture that does not require a predefined size of the input images. Among others, these two features make the final solution more portable and compliant with fashion and design requirements, which are against the introduction of more sensors (e.g., depth and RGB) for the same functionality.

Summarizing, the main contributions of this work are the following:

- We propose a *fully-convolutional Siamese network* to tackle the face verification task. Thanks to this architecture, the framework is able to deal with input images with different sizes;
- The presented method relies only on depth maps and achieves state-of-the-art results, not requiring any pre-processing step, like facial landmark localization, nose tip detection, or face frontalization;
- The face verification and identification accuracy are tested on three different depth-based datasets, namely *Pandora*, *High-Resolution Range-based Face Database* (HRRFaceD), and *Curtinfaces*;
- We design the Siamese network in order to have low memory requirements and real-time performance even on embedded platforms. We conduct an extensive speed performance evaluation of the proposed system on three different GPUs: the *NVidia 1080 Ti*, the *NVidia Quadro k2200*, and the embedded board *NVidia TX2*;
- Finally, through an experimental analysis that exploits the annotations of the *Pandora* dataset, we investigate the capabilities of the proposed approach and the influence of the head pose and visual occlusions on the face verification task.

Experimental results confirm the effectiveness of our approach that achieves state-of-the-art performance, relying only on depth data, on *Pandora*, *HRRFaceD*, and *CurtinFaces*.

## 2. Related Work

In order to guarantee a better comprehension of this work, we report here a survey about face recognition methods that rely on both intensity and depth data. Furthermore, we present and analyze the competitors for the proposed method that we use in this paper.

### 2.1. Face Recognition on Intensity Images

As stated before, the majority of literature works is based on intensity images: in this way, they tend to be sensitive to variations in pose, illumination, and expression changes [22,23]. Before the deep learning era, a variety of hand-crafted features were proposed [24,25,26,27,28,29,30]. However, the best results have been achieved through deep learning-based models [12,13,31]. In particular, in [12], a deep Siamese architecture was proposed. The input images were pre-processed through an alignment and a frontalization step and then fed into the network, which produced a similarity score. Schroff et al. in [13] proposed the use of a triplet loss on a face embedding space to cluster faces that belong to the same identity. The network, namely *Facenet*, achieved state-of-the-art results at the time of publication. In [32], an SVM classifier trained on visual attributes, e.g., age, gender, and ethnicity, was employed to learn a similarity score between faces. Siamese architectures were exploited by other works [33,34,35,36]. The main drawbacks of extremely deep networks are represented by the requirements in terms of training data and computational load (during both training and testing) and by the need to define a threshold value to discriminate between identities.

Recently, a well-established line of research has consisted of incorporating margins in loss functions. In [37], an *additive angular margin loss* was proposed, in order to obtain highly-discriminative features for face recognition. An extensive experimental evaluation was then proposed exploiting ten face recognition benchmarks based on intensity images. Similarly, in [38], a learned *cluster-based large margin local embedding* and a k-nearest cluster algorithm were combined, obtaining significant improvements over existing methods on both face recognition and face attribute prediction. In [39], the authors proposed to distance the representations of the identities through an exclusive regularization to obtain more discriminative features.

### 2.2. Face Recognition on Depth Maps

Thanks to the recent introduction of high-quality, but inexpensive depth sensors, like the *Intel RealSense* family or the *Microsoft Kinect* series, the interest of the research community in depth image processing and analysis has increased more and more in the last few years. Despite the lack of large-scale depth-based datasets in the literature, depth maps, also called range or 2.5D images, and 3D data (point clouds) are a valid kind of data for many computer vision problems that require working in the presence of dramatic light changes and darkness. Depth devices are based on three different technologies, i.e., *stereo cameras*, *structured light*, and *time-of-flight*, each of them with its pros and cons [40].

Depth maps have been employed in several face recognition methods. For instance, a *Pegasos SVM* [41] was used in [18] in order to tackle the one-vs.-all face identification task. The classifier relied on a modification of the well-known Local Binary Pattern (LBP) visual descriptors, referred to as *Depth Local Quantized Patterns* (DLQP). This method was improved in [42] through a new visual descriptor, called *Bag of Dense Derivative Depth Patterns* (Bag-D3P). Both methods assumed that all subjects were known during the training phase, and both were based on the dataset called *High-Resolution Range-based Face Database* (HRRFaceD), collected by the authors of the works. Recently, a deep Siamese architecture that is able to verify if two given faces belong to the same subject or not was proposed [21]. The architecture, called *JanusNet*, exploits the *privileged information* paradigm (also referred to as *side information*), in which some information is provided only during the training time in order to improve the performance of the system in the testing phase. This method, even though it improves the final performance, introduces a two-step complex training procedure, with a high number of loss functions. Moreover, during the testing phase, the input data need to be forwarded through two Siamese neural networks (which share the architecture, but have different weights), doubling the computational load with respect to a single Siamese network.

Other literature works were based on facial 3D models or exploited depth maps to build them. In [43], a transfer learning technique was proposed in order to train a CNN on 2D face images and to test it on 3D facial scans, after a fine-tuning phase with a limited number of point clouds. Besides, a face augmentation technique was proposed to synthesize a number of different facial expression from single facial scans.

Lee et al. [44] proposed a pipeline consisting of depth image recovery, feature extraction through a deep learning-based approach, and joint classification in order to recognize faces based on both color and depth information. In this procedure, facial landmark detection and face alignment tasks were strictly required.

In [45], a CNN for the face recognition task was proposed, based on low-level 3D local features (3DLBP) extracted from depth maps.

Differently from [21], we propose a method based on depth maps only (see Table 1 and Table 2 for details). Since we adopt a fully-convolutional Siamese architecture, the framework is able to deal with input images of different sizes, provided that the two branches of the Siamese network receive input images with the same spatial resolution. However, the best results are usually obtained using test images with a resolution as similar as possible to the ones of the training dataset. In addition, no specific pre-processing steps are required in order to train or test the network. Finally, as shown in Table 2, the adopted architecture relies on a limited number of parameters and is able to achieve higher accuracy in conjunction with better speed performance, despite the higher number of GFlops.

## 3. Method

In this section, we report the details about the proposed system, which is based on a fully-convolutional Siamese network, and the adopted training procedure. As stated before, the presented method does not require pre-processing steps, as is usually done by other works tackling the face recognition task, such as *face alignment* [46], *face frontalization* [47], or *facial landmark localization* [48]. The proposed approach only requires a face detection step, which is out of the scope of the presented work. Thus, we exploit dataset annotations to retrieve head-based cropped face images in absence of already cropped images, as detailed in the following sections.

### 3.1. Siamese Architecture

**Figure 1:**
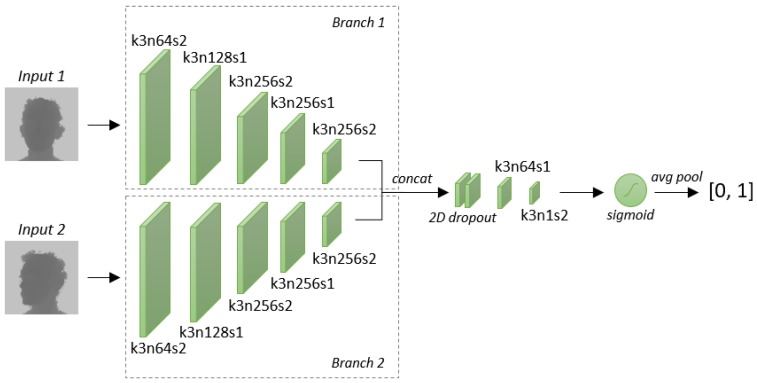
Overview of the proposed fully-convolutional Siamese architecture. The network takes a couple of facial depth maps as input and provides a continuous value in the range [0,1] as output, which is the probability that the input images belong to the same identity. Kernel size (*k*), number of filters (*n*), and stride size (*s*) are reported as the main parameters of each convolutional layer. With *concat* we refer to the concatenation on the feature channels.

The proposed architecture is depicted in Figure 1. The network is fed with a pair of depth images with a spatial resolution of 100×100 (since the proposed architecture is fully convolutional, this is not a hard constraint). Each Siamese branch has 5 convolutional layers, with 3×3 kernels and a stride of 2, except for the second and fourth layers that have a stride of 1. The number of feature maps increases along the architecture: the first and the second layer have 64 and 128 feature maps, respectively, while the following ones have 256 filters. Then, the outputs of the two branches are concatenated, and a 2D dropout is applied during training. Two additional convolutional layers with a 3×3 kernel size and 64 and 1 feature maps, respectively, constitute the final shared part of the architecture.

As suggested by several prior works [49,50,51], the *Rectified Linear Unit* (ReLU) activation function is employed after each convolutional layer, except for the last one, where the *sigmoid* activation is applied to output values in the range [0,1]. More precisely, the output of the last layer is a matrix of continuous values in the range [0,1], which is finally averaged to obtain a scalar value in the same range. The model is encouraged to predict a value near 1 when the two face images in the input belong to the same identity. Conversely, a value near 0 is expected when the input faces belong to different subjects.

### 3.2. Training Procedure

**Figure 2:**
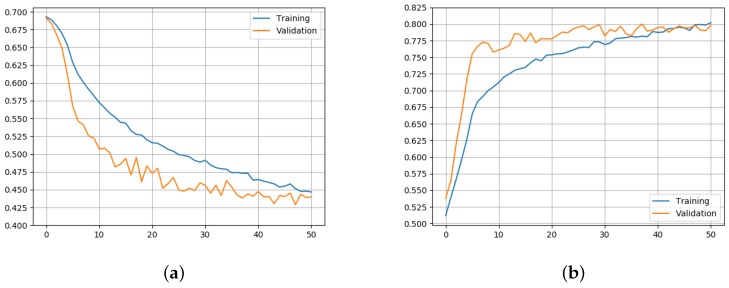
The graph on the left (**a**) shows the evolution of the loss function values during the training (blue) and validation (orange) steps. On the right, (**b**) reports the accuracy (expressed as a percentage in the range [0,1]) obtained during the training and validation phases.

We train the network using the *Stochastic Gradient Descent* (SGD) as the optimizer with a learning rate of 0.002, momentum of 0.9, and batches of 64 samples. We balance each mini-batch so that half of the samples has face pairs of the same identity, while the other half of the samples has face pairs of different subjects. We exploit the *batch normalization* [52] technique and the 2D dropout for regularization purposes. The standard *binary cross-entropy* loss function is used as the objective function:

(1) $$L=-\frac{1}{N}\sum_{i}\left(y_{i}\log\left(p_{i}\right)+\left(1-y_{i}\right)\log\left(1-p_{i}\right)\right),$$

where $y_{i}$ is the ground-truth value (i.e., 1 if the pair belongs to the same identity, 0 otherwise), $p_{i}$ is the predicted similarity, and *N* is the number of samples. In order to increase the reproducibility of the proposed approach, in Figure 2, we report the training and validation curves of the loss and accuracy values during the training process. As shown, the training phase converges after about 50 epochs.

## 4. Datasets

We tested the proposed architecture on three publicly-released datasets containing depth maps in addition to appearance images. In particular, the first one is pertinent to the automotive context.

### 4.1. Pandora Dataset

The *Pandora* dataset was presented in [17] and was created for training and testing systems for head pose estimation, since it contains the corresponding annotation. Several works exploited it for other related tasks, such as facial depth map generation [53], face detection on depth images [54], and attribute and landmark preservation [55].

The dataset consisted of both RGB and depth frames collected with the *Microsoft Kinect One* sensor (also called *Microsoft Kinect for Windows v2*), capturing the upper body part of the subjects. Data variety was obtained with the presentation of 22 subjects (10 males and 12 females); each subject was recorded 5 times for a total of 110 sequences. Thanks to the presence of many occlusions produced by garments and objects (e.g., smartphones, tablets) and extreme head and shoulder poses (±70° roll, ±100° pitch, and ±125° yaw), which dramatically affect the appearance of the subjects, the dataset is very challenging for the face verification task.

**Figure 3:**
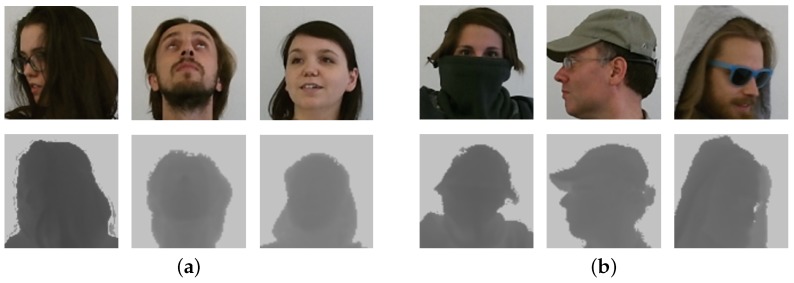
Sample depth and RGB frames taken from the Pandora [17] dataset. Frames from subsets $S_{1},S_{2},S_{3}$ are reported in (**a**), while frames from subsets $S_{4},S_{5}$ with garments and eye glasses are shown in (**b**). See Section 4.1 for further details about the subsets.

Similarly to [21], the sequences were split into two subsets. In the first one ($S_{1},S_{2},S_{3}$) were included sequences where only constrained movements were performed, i.e., pitch, yaw, and roll angles of the head and shoulder mainly varied one at time, while in the second one ($S_{4},S_{5}$), there were complex and free movements. Sample frames are reported in Figure 3.

Moreover, we defined three additional subsets taking into account the head angles. Referring to yaw, pitch, and roll angles as ρ,θ, and σ, the following three subsets were defined:

(2) $$A_{1}=\left\{s_{\rho \theta \sigma }\;|\;\forall \gamma \in \left\{\rho ,\theta ,\sigma \right\}:-10{}^{\circ }\leq \gamma \leq 10{}^{\circ }\right\},$$

(3) $$A_{2}=\left\{s_{\rho \theta \sigma }\;|\;\exists \gamma \in \left\{\rho ,\theta ,\sigma \right\}:\gamma <-10{}^{\circ }\vee \gamma >10{}^{\circ }\right\},$$

(4) $$A_{3}=\left\{s_{\rho \theta \sigma }\;|\;\forall \gamma \in \left\{\rho ,\theta ,\sigma \right\}:\gamma <-10{}^{\circ }\vee \gamma >10{}^{\circ }\right\}$$

**Figure 4:**
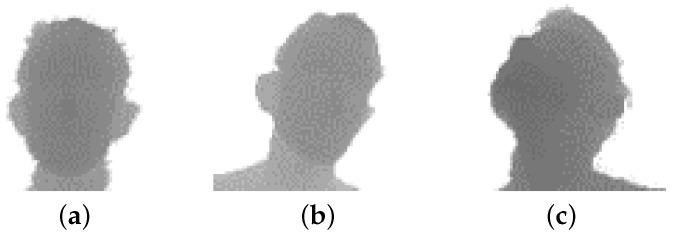
Sample frames taken from the subsets $A_{1}$ (**a**), $A_{2}$ (**b**), and $A_{3}$ (**c**) of the *Pandora* dataset, which contain frontal, non-frontal, and extreme head poses. See Section 4.1 for further details.

As a consequence, $A_{1}$ contained frontal head poses only, $A_{2}$ non-frontal faces, while $A_{3}$ all the faces with extreme head angles. Please note that $A_{3}$ was fully contained in $A_{2}$; thus, the three subsets were not disjoint. We conducted experiments using these subsets to investigate the influence of head poses and movements on the face verification task. We report an example of the extracted faces for each subset $A_{1},A_{2},A_{3}$ in Figure 4.

The total number of possible face pairs was very high (i.e., N2 unique pairs). Thus, in order to have a fair comparison with previous works, we adopted the same fixed set of image pairs proposed in [21].

The same validation set taken from the train set was used in order to stop the training when the validation accuracy was at the highest value.

Faces were cropped exploiting the annotations of the upper-body joints provided with the dataset. Given the frame coordinates *(x, y)* of the head centroid, referred to here as $\left(x_{H},y_{H}\right)$, we applied a dynamic crop in order to center the foreground, i.e., the subject’s face, and to include only a small part of background. The width $w_{H}$ and height $h_{H}$ of the bounding box around the face were defined as:

(5) $$w_{H}=\frac{f_{x}\;\cdot \;R_{x}}{D}\;h_{H}=\frac{f_{y}\;\cdot \;R_{y}}{D},$$

where $f_{x},f_{y}$ are the horizontal and vertical focal lengths, *D* is the distance between the acquisition device and the head center averaged on a square of 20×20 pixels, and $R_{x},R_{y}$ are the average width and height of a generic face, respectively. We set $f_{x}=f_{y}=365.337$ and $R_{x}=R_{y}=320$ in our experiments. The extracted bounding boxes were then scaled to 100×100 pixels.

### 4.2. High-Resolution Range-Based Face Database

**Figure 5:**
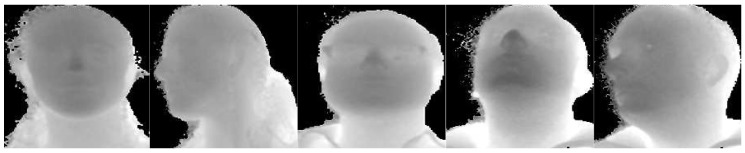
Sample depth images from the *High-Resolution Range-based Face Database* (HRRFaceD) dataset. As shown, heads were acquired under different poses.

The *HRRFaceD* dataset [18] consists of more than 20,000 images of 18 different subjects. It was collected with a *Microsoft Kinect One* sensor (the same device used to collect the *Pandora* dataset) placed at a distance of 50 cm from the faces. Male and female subjects were captured under different perspectives, and they extensively rotated their heads. We adopted the same splits reported in the original work for both the training and testing phase. Training and testing frames were samples from the same recording of each subject; as a consequence, the training and the test set were not subject-independent. Differently from *Pandora*, the *HRRFaceD* dataset provides already-cropped face images. Some sample frames taken from the dataset are reported in Figure 5.

### 4.3. CurtinFaces Database

The *CurtinFaces* dataset was released in [56], and it was specifically collected for the face recognition task under varying poses, expressions, illumination, and disguises.

**Figure 6:**
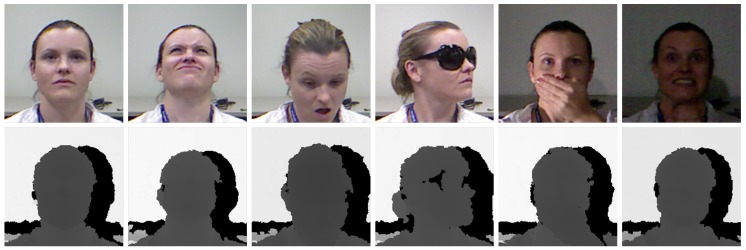
RGB and depth samples taken from the *CurtinFaces* dataset. As shown, different head poses, expressions, light variations, and garment occlusions are included.

It contains a limited amount of data (about 5000 images) consisting of both RGB and depth frames, acquired with the first version of the *Microsoft Kinect* sensor, a *structured light* depth device. For each of the 52 recorded subjects, 97 images are present. The first 3 images are the frontal pose and the left and right profile. Then, there are 84 images containing all possible combinations of 7 different poses and 7 expressions and all possible combinations of 5 different illumination variations and 7 expressions. Finally, 10 frames that include sunglasses and occlusions in different poses and illumination conditions are included. Sample images are shown in Figure 6. We cropped the face using the bounding box extracted with the well-known Viola and Jones detection algorithm [57]. The evaluation procedure reported in the original paper selected only 18 images per subject for the training phase, which included three variations in pose, expression, and illumination.

## 5. Results

We deeply investigated the performance of the proposed model using the three publicly-released datasets described above.

To increase the variety of training data, data augmentation techniques were applied. In particular, we applied random horizontal flip (probability 0.5) and a random rotation in the range of [−5,+5] degrees to the input images before providing them to the network. Thus, different images were used at each training epoch. Moreover, we tested our framework under different conditions, in terms of head poses and visual occlusions, in order to assess its robustness and capabilities. Finally, we also compared our approach with recent depth-based-only state-of-the-art methods [18,21,42].

### 5.1. Face Verification on Depth Maps

**Figure 7:**
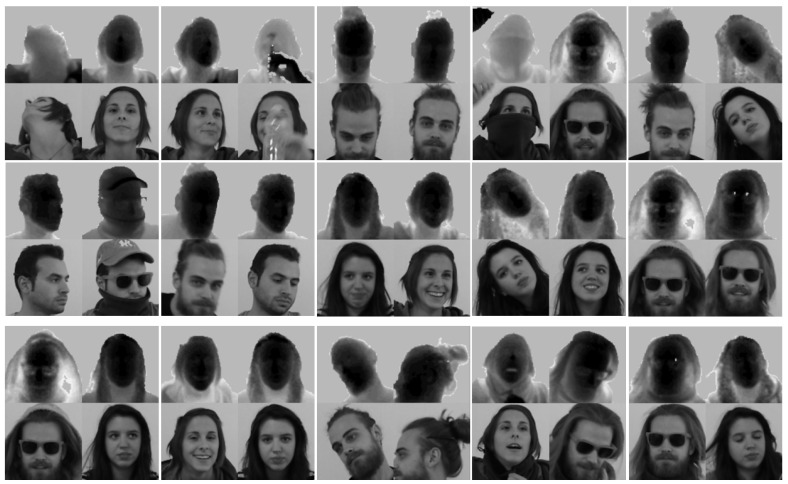
Sample output of the framework. From the top, the first block represents correct predictions, while wrong predictions are shown at the bottom. Depth maps are contrast stretched for a better visualization. Images were taken from the *Pandora* dataset.

**Figure 8:**
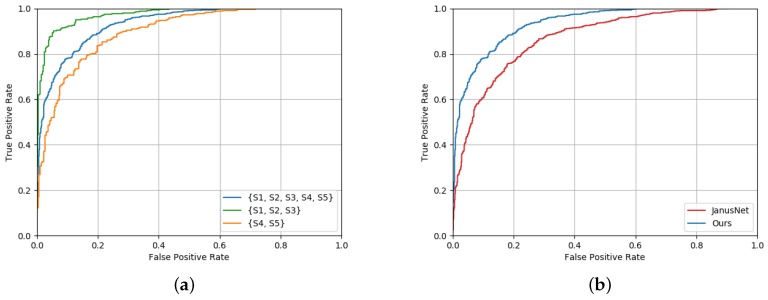
In graph (**a**), the ROC curves obtained by the proposed network computed on different splits of the *Pandora* dataset are reported (for further details, see Section 4.1). In (**b**), the ROC curves of the proposed method and the JanusNet architecture [21] are reported.

Results for face verification are reported in Table 3 and Figure 7. We observed that the proposed model achieved better results with respect to the *FaceNet*-like architecture based on RGB data, confirming that depth data and a shallow deep architectures are suitable for this task. Furthermore, the proposed model overcame the main competitor [21] in every setting. In particular, we report the results of the *JanusNet* architecture when trained with different kinds of data: only RGB, only depth, and both using the Privileged Information (P.I.) paradigm, which consists of adding knowledge at training time in order to improve the performance of the system in the testing phase. We also report the ROC curves of the proposed method and the state-of-the-art competitor [21] on the Pandora dataset in Figure 8. In particular, Figure 8a shows the ROC curves for different subsets of the Pandora dataset (for further details, see Section 4.1), while Figure 8b compares the ROC curves of our method and the one proposed in [21], showing the superior performance of the proposed approach.

### 5.2. Face Identification on Depth Maps

#### 5.2.1. HRRFaceD Dataset

Mantecon et al. [18,42] proposed a system for the face identification relying only on the depth data from the *HRRFaceD* dataset. To allow a comparison, we adapted the proposed face verification (*one-to-one*) system to tackle also the face identification (*one-to-many*) task. All the possible image pairs of the *HRRFaceD* dataset were fed to the Siamese network, producing a score value for each one. Since the dataset contained more instances of the same person, we developed and tested three different methods to select the final identity as proposed in [21].

Let $\xi (s,s^{\prime })$ be the similarity score between the couple of images $\left(s,s^{\prime }\right)$, as computed with the network described in Section 3.1. $S_{i}$ is the subset of images belonging to the *i*^th^ subject. We can define the following assignment functions:

(6) $$y_{max}=\underset{i}{arg max}\;\xi \left(s,s^{\prime }\right),\;\;\forall s^{\prime }\in S_{i}$$

(7) $$y_{avg}=\underset{i}{arg max}\;\underset{s^{\prime }\in S_{i}}{\mathrm{avg}}\xi \left(s,s^{\prime }\right)$$

(8) $$y_{voting}=\underset{i}{arg max}\;\#\left\{S_{i}\;\;|\;\;\xi \left(s,s^{\prime }\right)\geq t\right\},\;\;\forall s^{\prime }\in S_{i}$$

The final identity is the one with the highest absolute similarity using Equation (6) or the highest average similarity using Equation (7). A voting procedure is instead implemented in Equation (8): each pair with an output score greater than a threshold *t* votes for one identity, and the final identity is the one with the highest number of votes. In our experiment, we set t=0.75.

As reported in Table 4, the proposed model achieved high score performance and the best accuracy exploiting the *max* function, in line with the competitors. However, we observed that the *HRRFaceD* dataset was not challenging for the face identification task, since test frames were sampled from sequences included in the training split, thus making the test non subject-independent. In this way, the visual appearance of the faces in the training and the testing set was extremely similar, simplifying the task.

#### 5.2.2. CurtinFaces Dataset

We tested the proposed method on the *CurtinFaces* dataset also, relying only on the depth data. As in the previous case, we adapted the proposed model to tackle the face identification task, running the Siamese network on all the possible image pairs. We obtained the final score exploiting Equations (6)–(8) detailed above. Results are reported in Table 5. The first row of the table reports the accuracy obtained following the evaluation procedure detailed in the original paper (see Section 4.3) and used by the Sparse Representation Classifier (SRC) [56]. The proposed solution gave the best results. However, the adoption of this procedure led to an *over-fitting* phenomena, since the dataset contained a very limited amount of data. This hypothesis was confirmed by the fact that the overall accuracy increased by adding samples in the training split, as reported in the second row of the table.

### 5.3. How the Head Pose Influences the Face Verification Task

In real-world applications, face images are usually affected by occlusions caused, for example, by garments and hands. Moreover, the face can be partially visible due to lateral poses. An example is the automotive context, in which a hypothetical driver-monitoring system requires light invariance and high reliability to occlusions and movements. To this aim, we investigated how much the head pose influences the face verification task on the *Pandora* dataset splits described in Section 4.1. Results are reported in Table 6 and Table 7. Experiments reported in Table 6 confirmed that the face verification task was more challenging when extreme head poses occurred, and we note that the proposed model was able to handle severe rotations better than the competitor. The best results were achieved exploiting the subset $A_{2}$, which probably contained a more representative distribution of the *Pandora* dataset. Results also revealed that exploiting only images with extreme head poses (i.e., the subset $A_{3}$) partially compromised the training procedure, reducing the overall performance of the system. In the last row of the table, we report the test of our model trained on the whole dataset, which corresponds to the union of the subsets A1 and A2.

As shown in Table 7, the highest face verification accuracy was achieved on the subset $S_{1},S_{2},S_{3}$ while the subset $S_{4},S_{5}$, which contained strong visual occlusions produced by objects and garments, was the most challenging. As expected, a higher overall accuracy was obtained training the proposed network on the whole dataset.

### 5.4. Execution Time

We tested the execution time of the proposed system using as the input 100×100-pixel images extracted from the *Pandora* dataset. The model was developed and tested using the *PyTorch 1.1* framework [59]. The complete system had limited requirements in terms of memory usage: less than 1 GB of memory was used during the testing phase with a single batch size. Tests were conducted on three different settings.

The first one was a desktop computer equipped with an *Intel Core* i7-6850k processor and an *NVidia 1080 Ti* (3584 CUDA cores and 250 W of max power consumption). This setting was useful in order to test the proposed system without limitations in terms of power, memory, and energy consumption.

In addition, we employed a second desktop computer equipped with an *Intel Core* i7-950 processor and a *NVidia K2200* (640 CUDA cores and 68 W of power consumption), a self-powered GPU with limited memory and energy consumption.

Finally, we tested the framework on a *NVidia Jetson TX2*, an embedded system that integrates a GPU (256 CUDA cores) and a six-core processor (a combination of a quad-core *ARM Cortex-A57* and a dual-core *NVidia Denver 2*). With this test, we aimed to assess the inference time of the proposed face verification system on a board that could be easily integrated in a car or, in general, on UAVs and robots.

Results are reported in Table 3. The reported frames per second (fps) values were calculated by testing the inference time of the proposed architecture with a batch size of one (i.e., the inference time for one pair of images, averaged over 1000 iterations). As expected, the best results in terms of fps were achieved on the *1080 Ti*, but also the embedded board *TX2* achieved real-time performance (87 fps). Even if the GFlops required by our network were quite high with respect to the number of layers, the limited number of sequential layers with a high number of kernels resulted in a better parallelization of the process when running on GPUs and even on embedded boards such as the *NVidia Jetson TX2*. In particular, the presented approach overcame with a great margin all the competitors in terms of processed frames per second. Finally, the framework was able to run at 58 fps on an *Intel Core* i7-6850k CPU, without the help of the parallel computation of a GPU.

## 6. Conclusions

In this work, we proposed a fully-convolutional Siamese network to tackle the *face verification* task, relying only on depth maps.

We deeply investigated the performance of the presented model evaluating it on three public datasets, namely *Pandora*, *HRRFaceD*, and *CurtinFaces*. The shallow architecture effectively dealt with the limited size of depth-based datasets in the literature, while requiring low computational requirements, achieving real-time performance with a limited memory usage and state-of-the-art results on two public datasets. The feasibility and effectiveness of the proposed approach allowed the implementation in real-world challenging applications as, for instance, the automotive context that generally requires light-invariance and reliable algorithms.

In future work, we plan to acquire a new depth-based dataset in a realistic in-cabin environment to overcome the current lack of depth-based datasets collected in realistic conditions for the automotive context.

## Figures and Tables

**Table 1:** Summary of the main methods for *Face Identification* (FI) and *Verification* (FV) in the literature. In addition, for each method, we report the year of publication, the type and size of the input data, the pre-processing steps (Pre-proc.) required in terms of a priori required procedures (FA: *Face Alignment*, FL: *Facial Landmarks*, FF: *Face Frontalization*, FS: *Face Segmentation*), excluding the face detection and crop actions, since they are generally adopted by all the investigated methods.

Name	Year	FV	FI	Data Type	Input Size	Pre-proc.	Method (Features)
[12]	2014		✓	RGB	152×152	FA + FF	CNN
[13]	2015	✓	✓	RGB	224×224	-	Inception
[33]	2016	✓		RGB	100×100	FL + FA	CNN
[38]	2019		✓	RGB	112×96	-	CNN + CLMLE
[39]	2019		✓	RGB	112×96	FL + FA	ResNet20
[18]	2014		✓	Depth	180×180	-	Peg. SVM (DLQP)
[42]	2016		✓	Depth	180×180	-	Peg. SVM (Bag-D3P)
[45]	2017		✓	Depth	100×100	FS + FA	CNN (3DLBP)
[21]	2018	✓		RGB + Depth	100×100	-	Siamese CNN
Ours	2019	✓		Depth	variable	-	Siamese CNN

**Table 2:** Comparison between the proposed model and *JanusNet* [21]. Requirements in terms of input images, model parameters (number of weights, computational load, and final accuracy on the face verification task obtained on the *Pandora* dataset), and details on the embedded implementation are provided. Pros are highlighted in bold.

Method	Input Images			Model			Embedded Implementation		
	Input (Train)	Input (Test)	Input Size (∀ Branch)	#params ($10^{6}$)	#GFlops	Acc.	Weight Size	fps (CPU)	fps (TX2)
JanusNet [21]	RGB + Depth (paired)	Depth	100×100	4.8	**0.59**	81.4	18 MB	43	48
Ours	Depth	Depth	variable	1.8	0.73	85.3	7 MB	58	87

**Table 3:** Face verification accuracy on the *Pandora* dataset using the fixed test set, along with the computational requirements and the inference time, expressed as processed frames per second (fps). The proposed model is compared with every version of *JanusNet* [21], i.e., with different training data: only RGB, only depth, and both using the Privileged Information (P.I.) approach. We also report the performance of a *FaceNet*-like architecture for comparison.

Model	Data Type	Accuracy	#params ($10^{6}$)	#GFlops	fps${}_{1080\mathrm{Ti}}$	fps${}_{K2200}$	fps${}_{\mathrm{TX}2}$
JanusNet [21]	RGB	0.763	1.6	0.20	587	154	143
JanusNet [21]	Depth	0.795	1.6	0.20	587	154	143
JanusNet [21]	P.I.	0.814	4.8	0.59	202	50	48
**Ours**	**Depth**	**0.853**	**1.8**	**1.73**	**604**	**160**	**87**
FaceNet [13]	RGB	0.823	28.5	0.41	61	16	15
DenseNet [58]	-	-	28.7	7.82	45	13	11

**Table 4:** Accuracy comparison for the face recognition task on the *HRRFaceD* dataset. Functions *max*, *avg*, and *voting* are reported in Equations (6)–(8), respectively. In the last column, we report the Face Verification (FV) accuracy of the proposed method. The higher results are in bold.

	Pegasos SVM		JanusNet	Ours (FI)			Ours (FV)
	*DLQP*	*Bag-D3P*	*avg*	*max*	*avg*	*Voting*	*-*
**Accuracy**	0.735	0.943	0.987	**0.989**	0.985	0.959	0.981
**Improvement**	-	+20.9	+25.3	**+25.5**	+25.1	+22.5	-

**Table 5:** Face recognition accuracy on the *CurtinFaces* dataset. Functions *max*, *avg*, and *voting* are detailed in Equations (6)–(8), respectively. SRC refers to the *Sparse Representation Classifier* proposed in [56]. In the last column, we report the Face Verification (FV) accuracy of the proposed method. The higher results are in bold.

# Training Images		SRC [56]	Ours (FI)			Ours (FV)
18	79	*(Depth Only)*	*max*	*avg*	*Voting*	*-*
✓	-	0.887	**0.899**	0.857	0.817	0.860
-	✓	-	0.997	0.997	**0.998**	0.987

**Table 6:** Face verification accuracy on the *Pandora* splits. As reported in Section 4.1, the $A_{1}$ subset contains frontal head poses only, while $A_{2}$ and $A_{3}$ contain non-frontal poses, including extreme angles (up to ±70${}^{\circ }$roll, ±100${}^{\circ }$pitch, and ±125${}^{\circ }$yaw [17]).

	JanusNet 21]				Ours			
Train/Test	$A_{1}$	$A_{2}$	$A_{3}$	$\left\{A_{1},A_{2}\right\}$	$A_{1}$	$A_{2}$	$A_{3}$	$\left\{A_{1},A_{2}\right\}$
$A_{1}$	0.802	0.660	0.618	0.689	0.863	0.745	0.709	0.772
$A_{2}$	0.834	0.786	0.766	0.795	0.873	0.841	0.810	0.851
$A_{3}$	0.505	0.503	0.504	0.500	0.752	0.706	0.672	0.717
$\left\{A_{1},A_{2}\right\}$	0.798	0.751	0.727	0.762	0.885	0.842	0.812	0.853

**Table 7:** Face verification accuracy on the *Pandora* splits. As reported in Section 4.1, $S_{1},S_{2},S_{3}$ sequences contain constrained movements, while subsets $S_{4},S_{5}$ consist of complex movements and occlusions.

	JanusNet [21]			Ours		
Train/Test	$\left\{S_{1},S_{2},S_{3}\right\}$	$\left\{S_{4},S_{5}\right\}$	$\left\{S_{1},S_{2},S_{3},S_{4},S_{5}\right\}$	$\left\{S_{1},S_{2},S_{3}\right\}$	$\left\{S_{4},S_{5}\right\}$	$\left\{S_{1},S_{2},S_{3},S_{4},S_{5}\right\}$
$\left\{S_{1},S_{2},S_{3}\right\}$	0.844	0.746	0.773	0.888	0.780	0.817
$\left\{S_{4},S_{5}\right\}$	0.792	0.713	0.743	0.868	0.805	0.829
$\left\{S_{1},S_{2},S_{3},S_{4},S_{5}\right\}$	0.805	0.732	0.762	0.905	0.825	0.853
